# Functional aligned mesenchymal stem cell sheets fabricated using micropatterned thermo-responsive cell culture surfaces

**DOI:** 10.1016/j.mtbio.2025.101657

**Published:** 2025-03-10

**Authors:** Kenichi Nagase, Hasumi Kuramochi, David W. Grainger, Hironobu Takahashi

**Affiliations:** a Graduate School of Biomedical and Health Sciences, Hiroshima University, 1-2-3 Kasumi, Minami-ku, Hiroshima City, Hiroshima, 734-8553, Japan; b Faculty of Pharmacy, Keio University, 1-5-30 Shibakoen, Minato, Tokyo, 105-8512, Japan; c Department of Biomedical Engineering, University of Utah, Salt Lake City, UT, 84112, USA; d Cell Sheet Tissue Engineering Center (CSTEC), Department of Molecular Pharmaceutics, University of Utah, Health Sciences, Salt Lake City, UT, 84112, USA; e Institute of Advanced Biomedical Engineering and Science, Tokyo Women's Medical University, 8-1 Kawada-cho, Shinjuku, Tokyo, 162-8666, Japan

**Keywords:** Regenerative medicine, Stromal cell, Stem cell, Cell therapy, Transplantation, Tissue repair, Immunomodulatory, MSC secretome

## Abstract

Mesenchymal stem cells (MSCs) are frequently applied for cell transplantation and regenerative therapy because they secrete diverse therapeutic cytokines that prompt immuno-stimulatory and tissue repair processes. Furthermore, cultured MSC sheets exhibit enhanced cytokine secretion compared to their MSC suspensions, and represent a durable, versatile format for tissue engineering as singular, multi-layered, or multi-cell type sandwiched, transplantable constructs. Tissue engineered implants with various cellular orientations have been reported. In this study, patterned, temperature-responsive culture surfaces were used to prepare oriented MSC sheets. Patterned culture surfaces were fabricated by grafting polyacrylamide (PAAm) onto commercial poly(*N*-isopropylacrylamide) (PNIPAAm)-modified plastic via photopolymerization using a stripe-patterned photomask. Patterned surfaces were characterized using x-ray photoelectron spectroscopy, fluorescently labelled fibronectin and albumin adsorption assays, wetting (contact angle) measurements, atomic force microscopy, and scanning electron microscopy. Striped grafted patterns of PAAm were fabricated on the PNIPAAm-coated culture substrates, and PAAm polymerized within the PNIPAAm overlayer. Cell-aligned MSC sheets were then produced from MSC culture on this patterned surface, secreting higher amounts of therapeutic cytokines (vascular endothelial growth factor, hepatocyte growth factor, and transforming growth factor-β) than non-aligned MSC control sheets. In addition, aligned MSC sheets maintained enhanced cell multi-potent differentiation capabilities. New, aligned MSC sheets might exhibit improved functional properties for cell sheet transplant therapies.

## Highlights

• Stripe patterned culture surfaces were fabricated via photopolymerization.
• Patterned culture dish fabricates aligned mesenchymal stem cell sheet.
• Aligned mesenchymal stem cell sheet secrets higher amounts of therapeutic cytokines.
• The stem cell sheet would be applicable for effective cell sheet transplant therapies.

## Graphical abstract

**Figure:**
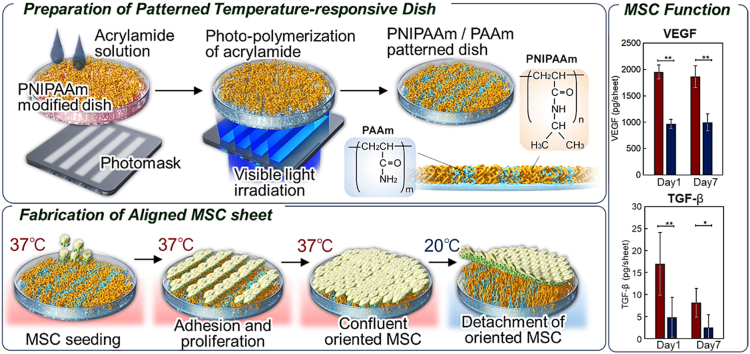

## 1 Introduction

Mesenchymal stem cell (MSC) therapies have been extensively investigated to treat diverse disorders. Transplanted MSC therapy is based on two primary modes of action: direct tissue replacement/repair via tri-lineage differentiation, and modulation of host cell activity via paracrine signaling and to a lesser degree, cell-cell contact signaling [[1], [2], [3]]. MSCs secrete myriad cytokines involved in cell proliferation, angiogenesis, inflammatory suppression, and immunoregulation (i.e., the MSC secretome) [[4], [5], [6], [7], [8]]. Most MSC transplantation exploit intravenous or catheter-based delivery of single-cell MSC suspensions. Thus, MSC biodistribution after infusion is not well controlled and MSC dosing to specific treatment sites relies on a homing mechanism that is not well understood and poorly validated in human therapy [9,10]. Injected MSC suspensions are generally plagued by poor MSC localization to and retention at disease sites [11], reducing sustained MSC dosing impact and escalating MSC numbers needed to treat humans [12].

Over 900 reported clinical trials have investigated MSCs for human therapy to date as shown on clinicaltrials.gov [[13], [14], [15]]. Safety of both autologous and allogeneic MSCs has been thoroughly demonstrated [[16], [17], [18], [19]]. However, MSC clinical efficacy is limited, as evidenced by the reduction in Phase II studies (250) to only 14 in Phase III (clinicaltrials.gov). After two decades of regulatory-compliant clinical trials and extensive peer-reviewed research, only three approved MSC therapies remain on the market for immune-related diseases globally, and notably, only one is just recently FDA-approved in the United States [20,21], and all with expensive pricing as customized therapies. Clearly, new insights into these long-standing challenges of bringing MSC therapies to address human disease are required.

To improve MSC therapeutic impact, MSC sheets have been investigated as an alternative to single cell infusion [[22], [23], [24], [25]]. MSC sheets have been conventionally prepared using commercial poly(*N*-isopropylacrylamide)(PNIPAAm)-modified cultureware. Given well-recognized temperature-responsive polymer conformational changes in aqueous milieu [[26], [27], [28], [29], [30]], PNIPAAm is exhaustively investigated for many different biomedical applications, including drug delivery systems [[31], [32], [33], [34], [35]], biosensor [[36], [37], [38], [39], [40]], bioanalysis and diagnostic system [[41], [42], [43], [44], [45], [46], [47], [48]], bioseparation systems [[49], [50], [51], [52], [53], [54], [55], [56], [57], [58], [59], [60], [61], [62], [63], [64]], and tissue culture substrates [[65], [66], [67], [68], [69]]. MSCs seeded on PNIPAAm-modified cultureware at 37 °C adhere to the culture surface because PNIPAAm is dehydrated and collapsed under these conditions, and MSC spontaneously adhere to this surface in culture. After 4–5 days under these conditions, MSCs have proliferated to reach confluence. Reducing culture temperature to 20 °C spontaneously changes PNIPAAm chain conformations under favored hydration, detaching adherent MSC sheets from the surface without conventional enzymatic treatment. MSC sheets prepared using PNIPAAm surfaces maintain better phenotypic profiles than cultured MSC suspensions prepared using digestive enzymes [70,71]. MSC sheets retain functional cell–cell junctions and their endogenous extracellular matrix (ECM). MSC sheets also exhibit higher engrafted survival rates after in vivo transplantation than MSC suspensions [72].

Aligned cell sheets have been developed previously using select types of temperature-responsive cell culture substrates. Striped patterns of fibronectin-printed PNIPAAm-modified culture plastic were prepared using microcontact printing and used to fabricate aligned sheets containing vascular smooth muscle cells [73]. In addition, microtextured temperature-responsive substrates presenting large arrays of alternating grooves and ridges (50 μm wide, 5 μm deep) were prepared by hot embossing polystyrene sheets and subsequent PNIPAAm grafting via electron beam irradiation [74]. Aligned smooth muscle cell sheets produced on these grooved surfaces maintained cell alignment after transfer from patterned culture to non-patterned culture substrates [74].

In another approach, grooved PNIPAAm film was prepared to align murine skeletal muscle C2C12 cells [75]. PNIPAAm/poly(acrylic acid-graft-azidoaniline) (PAA-*g*-Az) solution was placed on a polyethylene terephthalate film and then embossed with a grooved PDMS mold with grooved patterns (ridges/grooves 800 nm in width and 500 nm in depth) [76] and fabricated using electron beam lithography and dry etching techniques [77]. The grooved PNIPAAm film was then exposed to UV light for cross-linking and used to prepare aligned C2C12 cell sheets.

Stripe-patterned polymer-brush-grafted glass was prepared by RAFT polymerization and the subsequent stripe-pattern grafting of hydrophilic poly(*N*-acryloylmorpholine)(PAcMo) [[78], [79], [80]]. Stripe-patterned block copolymer brushes of PNIPAAm and PNIPAAm-*b*-PAcMo were used to create aligned dermal fibroblast sheets exhibiting elevated cytokine secretion, and also myoblast sheets exhibiting myotube formation [[78], [79], [80]].

Aligned MSC sheets are not yet reported and represent a rational approach for augmenting their therapeutic potential. Reliable production of aligned MSC sheets could be used to improve their cytokine secretion profiles, possibly enhancing their therapeutic utility.

We report unique patterned MSC sheets enabled by analogous temperature-responsive cell culture methods on grooved, aligned substrates. Various stripe-patterned temperature-responsive cell culture substrates were prepared by masked photopolymerizing polyacrylamide (PAAm). The prepared PNIPAAm patterned dish can be prepared using commercially available PNIPAAm cell cultureware with simple surface modifications using PAAm without nano-fabrication procedures. Furthermore, PAAm remains covalently bonded to the dish surface, resulting in high pattern stability. Aligned MSC sheets were characterized and shown to exhibit properties distinct from non-aligned MSC sheets.

## 2 Materials and methods

### 2.1 Preparation of patterned temperature-responsive cell culture substrates

**Fig. 1:**
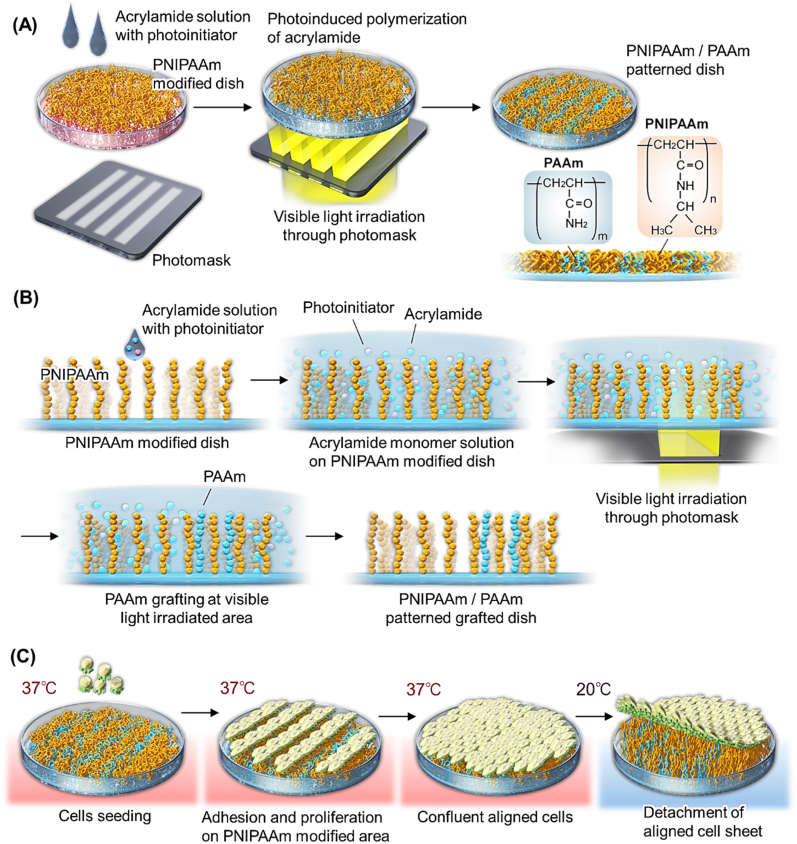
Schematic illustration of (A) preparation of patterned cell culture substrates through patterned photopolymerization of acrylamide onto commercial thermo-responsive cell culture plastic, (B) schematic of selective surface pattern modification using acrylamide, and (C) cultured cell alignment yielding oriented cell sheets using patterned culture surfaces.

Patterned temperature-responsive culture dishes were prepared by photopolymerization of acrylamide into varying line patterns on PNIPAAm cell culture surfaces (see Fig. 1). All reagents used to prepare patterned temperature-responsive culture dishes are described in Supplementary Materials.

Acrylamide (2.50 g, 35.2 mmol) and 7,7-dimethyl-2,3-dioxobicyclo [2.2.1] heptane-1-carboxylic acid, and photo-initiator camphorquinone (50.0 mg, 0.255 mmol) were dissolved in pure water (2.5 mL). Two adhesive plastic films (20 × 2 × 0.1 mm) were affixed to the inner surface of commercially available temperature-responsive cultureware (UpCell™, CellSeed, Tokyo, Japan) in parallel configuration with a distance of 20 mm between the two films. A 23 mm square cover glass was covered with two films, resulting in a 0.1 mm thick space on the cultureware surface. This gap was then filled with the prepared acrylamide monomer solution (50 μL). The culture substrate was then placed bottom side up covered with a photomask and further completely covered with a glass plate. Visible light was applied for 5–7 min (Fig. 1). Three types of lined photomasks were used with alternating stripe micron-sized widths as follows: 100 μm/50 μm, 50 μm/50 μm, and 50 μm/20 μm (widths of non-transparent and transparent areas of visible light, respectively) (Fig. S1). After visible-light irradiation, the cover glass was removed, and the culture substrate was washed with ultrapure water to remove non-adherent monomer and polymer. In addition, these culture dishes were filled with ethanol, washed for 18 h on a shaker to completely remove PAAm solution, and dried on a clean bench for 30 min. Prepared patterned substrates were each named using the first letter of each monomer (NIPAAm =N and AAm = A) and ratio of the line pitch. For example, a patterned dish with a patterned polymer ratio of PNIPAAm to PAAm = 50:50 was “NA50:50”.

To avoid cell detachment during long-term culture required to differentiate MSC sheets, patterned PAAm-modified TCPS culture dishes were prepared because these patterned PAAm-modified TCPS surfaces yielded relatively strong MSC adhesion compared to that for patterned PAAm-modified PNIPAAm dishes.

### 2.2 Characterization of patterned temperature-responsive cell culture dish

Patterned temperature-responsive culture dishes were characterized by x-ray photoelectron spectroscopy (XPS: JPS9010TR; JEOL, Tokyo, Japan), take-off angle of 15°, protein adsorption, attenuated total reflection/Fourier transform infrared spectroscopy (ATR/FT-IR: Nicolet 6700, Thermo Fisher Scientific, Waltham, USA), water contact angle meter (DSA 100S, Kruss, Hamburg, Germany), atomic force microscopy (AFM: AFM5100N, Hitachi High-Tech, Tokyo, Japan), and scanning electron microscopy (SEM: MERLIN Compact; Zeiss, Baden-Wurttemberg, Germany).

Prepared patterned temperature-responsive culture dishes, commercial temperature-responsive culture dishes, and standard tissue culture polystyrene were cut into 10 mm × 10 mm pieces. The surface elemental composition of the cut pieces was analyzed using XPS (collecting low and high resolution XPS data for elements, C, O, and N).

Photopolymerized patterns were interrogated using fluorescently labelled protein adsorption. Rhodamine-labelled bovine fibronectin (10 μg) was dissolved in 1 mL of phosphate buffered saline (PBS), placed in the patterned culture dishes, and then incubated at 37 °C for 2 h. Alexa488-conjugated BSA (10 μg) was dissolved in 1 mL PBS, added to patterned culture dishes, incubated at 37 °C for 2 h, then rinsed with PBS. After incubation, both dishes were dried and observed under fluorescence microscopy (ECLIPSE TE2000-U; Nikon, Tokyo, Japan). PNIPAAm and PAAm content in patterned dishes was analyzed using ATR/FT-IR spectroscopy using a Ge ATR accessory (VariGATR, Harrick Scientific Products, Pleasantville, NY, USA) with 128 scan and 0.964 cm^−1^ resolution under air. Surface wettability of the prepared patterned dish was investigated using aqueous contact-angle measurements. A water droplet (10 μL) was dropped onto the surface at eight locations and the static contact angle of the droplet under ambient air was measured on both sides of the sessile droplet at 37 °C. Surface morphology of patterned dishes was assessed using AFM (AFM5100N, Hitachi High-Tech, Tokyo, Japan). The dry surface of a patterned culture dish imaged in dynamic force mode (DFM) under ambient air over a scan range (100 μm × 100 μm and 50 μm × 50 μm). Patterned dish surfaces were also observed using SEM.

### 2.3 Preparation and analysis of aligned human dermal fibroblast (NHDF) and MSC sheets

All cell culture media, reagents, and cell culture protocols are described in Supplementary materials.

Normal human dermal fibroblast (NHDF) sheets were prepared as follows: NHDF cell suspensions in NHDF culture medium (2 mL at 4.0–4.5 × 10^4^ cells/mL) were seeded onto patterned and non-patterned cultureware and incubated at 37 °C for 3–4 days until NHDF reached confluence. Dishes were then incubated at 20 °C for 30 min, detaching NHDF sheets which were then analyzed.

Mesenchymal stem cell (MSC) sheets were prepared as follows. Human bone marrow-derived MSC suspensions prepared using MSC culture medium at 4.0–4.5 × 10^4^ cells/mL (2.0 mL) were seeded onto patterned dishes. Dishes were incubated at 37 °C for 3–4 days until MSCs proliferated to confluence. Dishes were then incubated at 20 °C for 30 min until MSC sheets spontaneously detached.

Sheet cell density was measured by subsequent sheet incubation for 30 min in 2.5 g/L-trypsin/1 mmol/L-EDTA solution (1 mL) to dissociate the cell-cell connections and produce single cells. Next, 2 mL of fibroblast or MSC culture medium was added to the dish, and the resulting cell suspension was centrifuged at 220×*g* for 5 min. After centrifugation, the supernatant was removed and 1 mL of culture medium was added. The cell suspension (10 μL) was collected, stained with trypan blue, and cell numbers were counted using a blood cell calculator.

The degree of cell alignment in both cell sheets was determined using the following procedure: a phase contrast microscope (BZ-X810; Keyence, Osaka, Japan) was used to photograph the adherent cell sheet as cultured on patterned or unpatterned culture surfaces. Phase-contrast images were analyzed using ImageJ (NIH, Bethesda, USA) to produce quantification of cell alignment and extracting data on overall cell orientation.

Select secreted cytokines from NHDF sheets and MSC sheets were measured using ELISA. Cell culture medium over confluent NHDF sheets on patterned dishes was removed, and fresh NHDF culture medium (1 mL) was added to the dish and incubated at 37 °C for 1 day. Culture media in the dish was then collected and cytokines in the medium were measured using commercial ELISA kits specific to each cytokine. This medium was defined as Day 1. To investigate longer-term cytokine expression, more cell culture medium was added to dishes and cell sheets were incubated for 5 days. Cell culture medium was subsequently changed and further incubated for 24 h. Culture media were then collected from each dish and defined as Day 7. Cytokines in culture medium samples collected on Days 1 and 7 were measured using an ELISA kit for each cytokine.

MSC sheet differentiation ability was investigated using the following procedure. MSC detachment during longer-term culture was hindered by using patterned PAAm striped on conventional TCPS cell culture dishes. Patterned PAAm on TCPS exhibits stronger cell adhesivity compared to PAAm stripes on PNIPAAm cultureware because TCPS has stronger cell adhesivity compared to PNIPAAm cultureware. MSC were seeded onto patterned TCPS dishes at a density of 1.8 × 10^4^ cells/cm^2^ and incubated at 37 °C for 4 days. After MSCs reached confluence, the cell culture medium was removed, osteogenic differentiation medium (2 mL) was added, and the dish was incubated for 14 days, changing osteogenic differentiation medium every 3 days. Alizarin Red S staining was then performed to analyze MSC osteogenic differentiation. MSC sheet adipogenic differentiation involved the same procedure, except that adipogenic differentiation medium in place of osteogenic differentiation medium. Oil Red O staining was performed to assess MSC adipogenic differentiation.

### 2.4 Statistical analysis

All values are expressed as average values and standard deviations (mean ± SD). Differences were analyzed using Student's t-test for two groups and one-way ANOVA test for more than three groups. Statistical significance was set at P < 0.05.

## 3 Results and discussion

### 3.1 Characterization of pattered temperature-responsive culture substrates

Photo-patterned stripes on cell culture surfaces were characterized by XPS, protein adsorption, ATR/FT-IR, wetting contact angle measurements, AFM, and SEM.

**Fig. 2:**
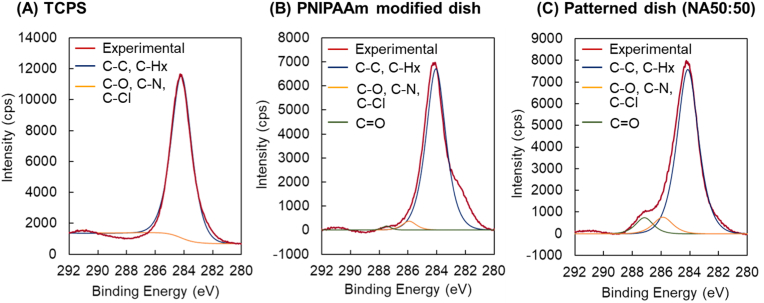
High-resolution XPS C1s peak deconvolution for (A) TCPS, (B) PNIPAAm-modified culture substrate, and (C) PAAm-patterned culture substrate (take-off angle 15°).

Surface elemental composition of patterned dishes (both TCPS- and PNIPAAm-modified dishes) was investigated by XPS (Table 1). XPS C1s peak deconvolution for high-resolution spectra from TCPS, PNIPAAm-modified and PAAm-patterned dishes was conducted (Table 1 and Fig. 2). Predictably higher nitrogen and oxygen content is observed in PNIPAAm-modified surfaces compared to those of TCPS, attributed to nitrogen and oxygen composition of PNIPAAm grafted on the TCPS dish surface. C1s peak deconvolutions show a small high energy shoulder peak attributed to C=O and C-N attributed to the acrylamide chemistry of PNIPAAm, while no higher energy C1s peaks attributed to C=O and C-N are observed in the TCPS spectrum (Fig. 2). PAAm-patterned surfaces exhibit higher nitrogen and oxygen composition than PNIPAAm-modified surfaces consistent with PAAm's higher density of acrylamide side chains (see Table 1 theoretical C/N ratios). XPS data (Table 1 and Fig. 2) support distinct surface chemistries for polymer-modified TCPS versus TCPS alone, and importantly, successful PAAm modification on PNIPAAm surfaces via photo-polymerization.

**Table 1:** Comparative XPS surface elemental analyses of cell culture surfaces (take-off angle 15°).

Surface	Atom (%)			N/C ratio	C1s peak deconvolution (%)		
	C	N	O		C=O,	C-O, C-N, C-Cl	CHx,
					>N-C=O		C-C, C=C
TCPS	94.6	0.5	4.9	–	<1	1.0	99.0
PNIPAAm- modified surface	89.9	3.6	6.4	0.04	1.1	3.8	94.1
PAAm patterned surface NA50:50	80.0	9.0	11.0	0.11	6.7	7.7	85.6
Theoretical composition:							
PSta)	100.0	0.0	0.0	–			
PNIPAAma)	75.0	12.5	12.5	0.17			
PAAma)	60.0	20.0	20.0	0.33			

a ) theoretical atomic composition of PSt (untreated polystyrene), PNIPAAm, and PAAm (excluding hydrogen, H).

**Fig. 3:**
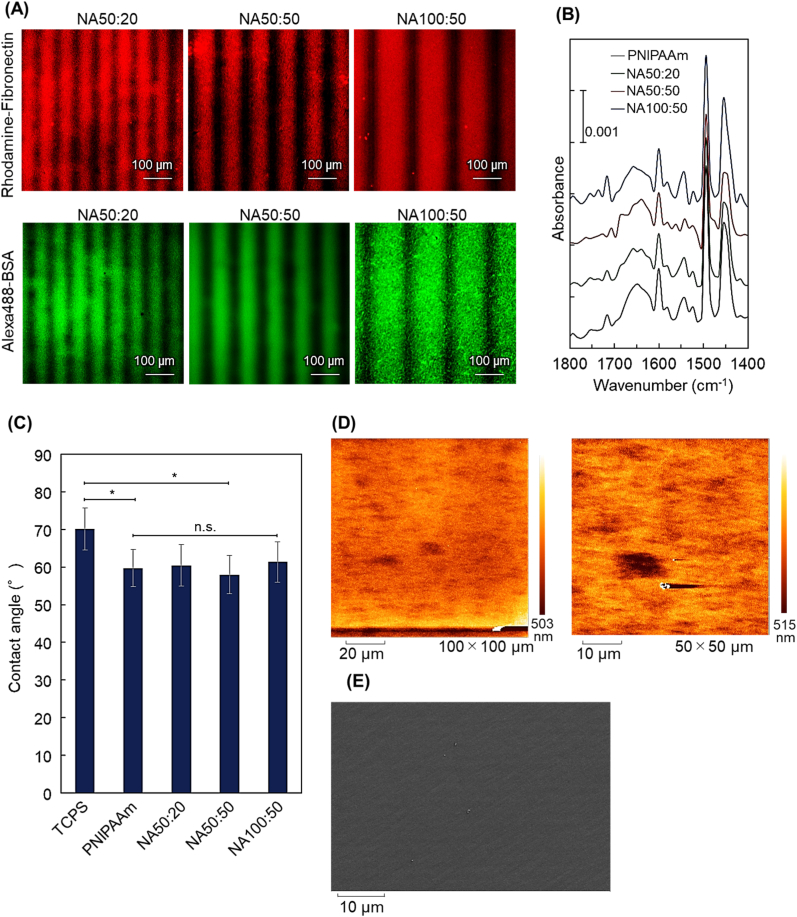
Characterization of PAAm-patterned commercial PNIPPAm-modified culture surfaces. (A) fluorescent microscopy images of adsorbed rhodamine-labelled fibronectin and Alexa488-labelled bovine serum albumin in patterns. (B) ATR/FT-IR vibrational spectra. (C) Wetting comparison: aqueous sessile drop contact angle measurements at 37 °C (∗*p* < 0.05; n.s.: not significant). (D) AFM tapping mode image. (E) SEM surface image.

Fluorescein-labelled fibronectin and BSA adsorption on PAAm-patterned surfaces was conducted to understand protein interfacial behaviors on these line patterns (Fig. 3A). Patterned fibronectin and BSA adsorption on line patterns corresponding to the substrate pattern line spacings were observed (Fig. 3A). This image contrast is attributed to reduced protein adsorption to PAAm versus PNIPAAm under these conditions in which more highly hydrated PAAm line chemistry is more protein resistant than more hydrophobic PNIPAAm. Selective and differential protein adsorption to the different surface chemistries indicates that the PAAm/PNIPAAm patterns produce patterned high and low protein adsorption areas that influence how seeded cells attach to these surfaces in serum-containing culture media.

PAAm-pattern surface modification was further investigated using reflectance ATR/FT-IR (Fig. 3B). A small intensity IR band (∼0.005 absorption units) was observed at 1650 cm^-1^ for three PAAm-patterned and PNIPAAm-modified dishes, consistent with amide bonds common to both PNIPAAm and PAAm. However, no obvious difference in this peak intensity (or other IR features) was observed to distinguish three PAAm-patterned surfaces from commercial PNIPAAm-modified surfaces. This was likely due to the relatively small amounts of PAAm surface modification both in stripe surface area and with depth, relative to the underlying PNIPAAm matrix dominating the penetrating ATR IR excitation field.

Aqueous surface wettability was investigated using sessile drop contact angle measurements at 37 °C (Fig. 3C). For comparison, contact angles for commercial PNIPAAm surfaces and TCPS were also measured. PNIPAAm dishes exhibited lower water contact angles than that from TCPS due to surface-modified, more polar PNIPAAm imparting higher hydrophilicity. Three PAAm-patterned dishes exhibited similar contact angles as the unmodified PNIPAAm dish surface. Amounts of surface-modified PAAm are quite small, and PAAm occupies roughly half or less the surface area in stripe patterns. Additionally, the abrupt, repeating PAAm-PNIPAAm striped alternating chemical and topological interface likely modifies the sessile probe drop 3-phase wetting equilibrium. Hence, the small contact angle changes seen can have several interpretations.

Surface topography was imaged using AFM and SEM (Fig. 3D and E). AFM and SEM images both showed no obvious differences in roughness or feature heights anticipated for 50 μm-wide line pattern modifications of PAAm on PNIPAAm. One explanation is that AAm monomer solution applied as lines during photopolymerization initially wets and absorbs into the PNIPAAm substrate and then polymerizes as lines within the PNIPAAm layer on the dish, not on top of this layer. The commercial PNIPAAm-modified culture surface is prepared by EB irradiation-induced polymerization of NIPAAm monomer onto TCPS [65,81], yielding sub-micron-thick PNIPAAm uniform overlayers on culture plastics. Thus, the AAm monomer solution permeates this PNIPAAm layer rather than residing on top of it. Thus, the PAAm-modified stripes do not produce an increase in height compared to the PNIPAAm base layer, but still influence local chemical composition, wetting and protein adsorption.

### 3.2 Fabrication and characterization of aligned cells in oriented NHDF sheets

**Fig. 4:**
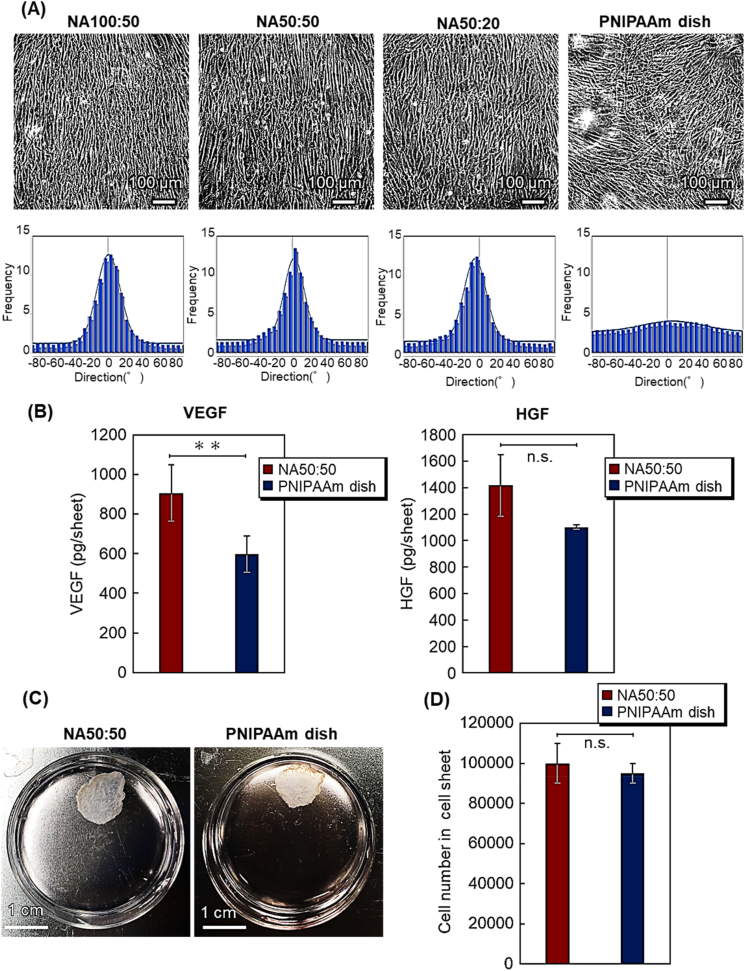
Characterization of aligned, versus non-aligned NHDF sheets. (A) phase contrast microscopy comparing aligned and non-aligned NHDFs in sheet cultures. (B) cytokine expression in aligned versus non-aligned NHDF sheets. (C) Images of NHDF sheet detachment from each surface by reducing culture temperature. (D) cell numbers in both types of cell sheets (∗∗*p* < 0.01; n.s.: not significant).

As an initial stromal cell model for inducing sheet alignment, NHDF sheets were fabricated in patterned dishes because previous reports showed that cell alignment to yield oriented NHDF sheets was possible with cell culture surfaces patterned in other ways [78,79]. Cells in NHDF sheets adhered on both patterned and non-patterned surfaces were directly observed with phase contrast microscopy (Fig. 4A). Aligned NHDF cells within sheets are seen on all PAAm-patterned dishes of different line widths (NA100:50, NA50:50, and NA50:20), whereas NHDF cells exhibit no preferred alignment or growth directions on PNIPAAm-modified dishes. NHDFs adhere to and then align and grow along stripe patterns, specifically or preferentially to the PNIPAAm-modified areas, and not to PAAm-modified areas, consistent with types and amounts of serum proteins adsorbed from culture media to each surface area that selectively promote (i.e., PNIPAAm) or discourage (i.e., PAAm) NHDF adhesion to these respective zones. NHDF cells elongate in registration with PNIPAAm-modified striped areas, and proliferate along the PNIPAAm-PAAm interfaces, avoiding PAAm areas. After NHDFs occupy PNIPAAm areas of the dish, they extend into PAAm-modified areas while maintaining alignment. NHDFs eventually create a confluent largely aligned cell monolayer in all patterned dishes (Fig. 4A). In contrast, NHDF seeded on unpatterned commercial PNIPAAm dishes adhere in various orientations, yielding non-aligned monolayers.

Histograms of final cell alignment/orientation generated from ImageJ analysis (Fig. 4A) show that the sharp narrow cell alignment distribution for the NA50:50 pattern contrasts those for NA100:50 and NA50:20 alignment patterns, indicating that 50 μm width patterned lines and spacings produce best spontaneous NHDF alignment. On NA100:50 patterns, NHDF initially adhered to the 100 μm wide PNIPAAm-modified area and then extend in broad angles of orientation because the 100 μm available area for extension is large compared to the NHDF cell size, producing now orientation stimulus. NHDF alignment on NA100:50 was therefore relatively low compared to that of NA50:50. In contrast, on NA50:20 patterns, NHDFs adhere to 50 μm PIPAAm areas and extend across the 20 μm-wide PAAm-modified stripes because the PAAm-modified area is narrow versus cell size. This yields broad angles of cell orientational distributions, leading to reduced NHDF sheet alignment.

Cytokine secretion from NHDF sheets was investigated using ELISA (Fig. 4B). Aligned NHDF sheets were prepared to confirm the influence of microfabricated patterned temperature-responsive cultureware on cell alignment-induced secretome enhancement, as reported previously in similar cell sheet studies using a different cell alignment method [79]. In the previous study, aligned NHDF sheets secreted larger amounts of vascular endothelial growth factor (VEGF) and hepatocyte growth factor (HGF), two cytokines recognized for synergistic pro-regenerative properties, also in cell sheets [[79], [82]]. For direct comparison of cell alignment effects, this study examined the same VEGF and HGF cytokine secretory behavior as in the previous study [79].

VEGF is increased and HGF is modestly increased in NHDF sheets cultured on NA50:50 patterned dishes compared to unpatterned PNIPAAm-modified dish sheets. Aligned NHDF sheets secrete larger amounts of cytokines compared to non-oriented NHDF sheets. This is attributed to enhanced cell functionality when NHDFs are oriented in dense-packed sheets, with a cell environment more similar to in vivo dermal tissue. Furthermore, cell alignment may yield stronger cell-cell adhesion, interactions and engagement in oriented cell sheets than in non-oriented cell sheets that prompts these cytokine enhancements [82]. Significantly, aligned NHDF sheets fabricated in this study demonstrated similar enhanced levels of cytokine secretion compared to oriented NHDF sheets fabricated in a previous report [79]. These results confirmed the effects of cultureware patterning on NHDF cell alignment and subsequent influences on cytokine production.

Cell sheet detachment was performed by temperature reduction, and resulting morphology of the detached cell sheets was observed (Fig. 4C). Stromal cell sheets have a profound spontaneous contraction once released from PNIPAAm culture surfaces that prompts cell cytoskeletal reorganization and measurable paracrine changes [82]. Released oriented NHDF sheets exhibit a more-oval sheet shape, whereas released non-oriented NHDF sheets are more circular. Elongated aligned NHDFs in oriented NHDF sheets contract along a similar vector likely guided by oriented cytoskeleton produced by sheet formation [82,83]. Non-aligned, non-oriented sheets contract as well but in no preferred direction or anisotropy. Physically this stromal cell sheet anisotropic contractile behavior is recently explained for NIH 3T3 fibroblast cell sheets aligned on striped elastomeric substrates as a change of order parameter: the sheet in-plane alignment determines the resulting anisotropic structure of the released cell sheet. Aligned sheet contraction is greater along their nematic director imposed by cultured cytoskeletal alignment than perpendicular to it, yielding non-circular anisotropic morphologies upon sheet detachment [84].

Cell numbers in released sheets was determined by dissociation of sheet cell-cell connections using trypsin and subsequent cell counting under trypan blue staining (Fig. 4D). No obvious differences in cell numbers are observed between oriented and non-oriented NHDF sheets. These results suggest that enhanced cytokine expression from aligned cells in oriented NHDF sheets was not attributed to different NHDF cell counts in sheets but to increased sheet-stimulated paracrine expression [82,85,86].

### 3.3 Fabrication and characterization of cell alignment in oriented MSC sheets

**Fig. 5:**
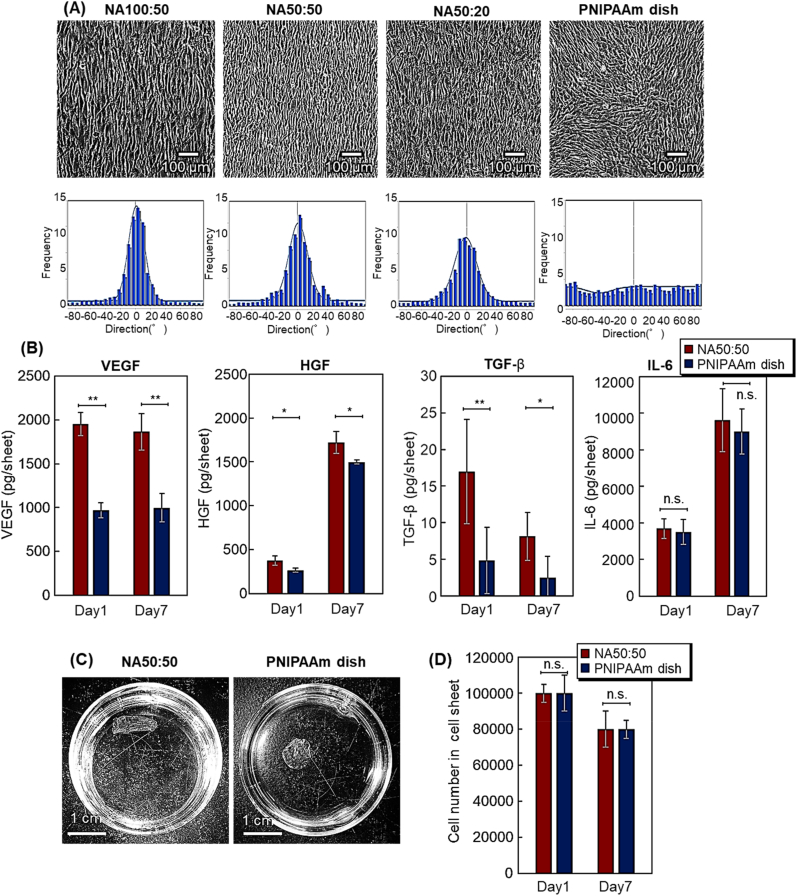
Characterization of aligned, oriented versus non-oriented MSC sheets. (A) phase contrast microscopy comparing aligned and non-aligned MSCs in sheet cultures; (B) cytokine expression in aligned versus non-aligned MSC sheets; (C) images of MSC sheet detachment from each surface by reducing culture temperature; (D) cell numbers in both types of cell sheets (∗*p* < 0.05; ∗∗*p* < 0.01; n.s.: not significant).

Cell alignment in oriented MSC sheets was produced using various patterned culture surfaces, and resulting MSC behaviors in the various MSC sheets was contrasted in Fig. 5A. MSC adhesion and anisotropic extension with consequent cell-cell orientation is observed on all patterned surfaces (NA100:50, NA50:50, and NA50:20), but not on commercial unpatterned PNIPAAm-modified dishes where random cell surface arrangement is seen. Similar to NHDFs (Fig. 4A), MSCs initially adhered to PNIPAAm-modified areas and extend, guided along the stripe pattern interface and proliferating within PNIPAAm zones parallel to pattern lines (Fig. 5A). After occupying PNIPAAm areas, MSCs then extend into PAAm-modified areas but maintaining their orientation. MSCs on patterned surfaces thereby yield adherent cell sheets with anisotropically aligned cells on patterned dishes. In contrast, MSCs adhered and extend on unpatterned commercial PNIPAAm-modified dishes to exhibit variable, more random placement without individual or overall sheet orientation.

Histograms of cell orientation (Fig. 5A) generated using ImageJ processing of these phase contrast images are consistent in supporting visual interpretations: NA100:50 and NA50:50 patterns produce relatively sharp cell alignment histograms compared to that for NA50:20. Unlike NHDFs (vida supra), MSCs on NA100:50 patterns yielded oriented MSC sheets (Fig. 5A). Pattern spacing of 100 μm wide:50 μm wide (cell-adhesion region:low-adhesion region) better accommodated both MSC size and extensibility. However, the NA100:50 pattern prompts pre-mature MSC sheet detachment during sheet culture, as seen in other MSC sheet culture conditions [83]. As NA50:50 reliably yielded oriented NHDF sheets, this culture pattern was selected for further MSC sheet investigation.

Cytokine secretion in culture-adherent MSC sheets was investigated (Fig. 5C). Since MSC sheets are intended for specific, diverse therapeutic applications, we examined the secretion of cytokines with known therapeutic effects from previous MSC secretome reports [[87], [88], [89], [90], [91]]. Substantially increased VEGF secretion was observed in cell-aligned, oriented MSC sheets compared to that from non-oriented MSC sheets. More modest, relatively higher HGF secretion is observed from oriented MSC sheets versus non-oriented MSC sheets. Furthermore, oriented MSC sheets secreted more transforming growth factor-β (TGF-β) versus non-oriented MSC sheets. In contrast, interleukin 6 (IL-6) secretion shows no significant differences between oriented and non-oriented MSC sheets. Higher secretion of VEGF, HGF, and TGF-β from oriented MSC sheets is attributed to elongated anisotropic MSCs having higher cytokine expression ability, as previously reported in various anisotropic MSC culture systems [92,93]. Furthermore, confluent MSC alignment may yield stronger cell-cell adhesion, interactions and engagement in oriented cell sheets than in non-oriented cell sheets that prompt these observed cytokine enhancements [82].

High VEGF secretion from aligned MSC sheets would be applicable to current MSC sheet transplantation therapy for ischemic heart disease yielding enhanced angiogenesis [87,88]. High HGF amounts secreted from aligned MSC sheets are attractive in treating liver diseases [89,90]. Aligned MSC sheets secreting large amounts of TGF-β1 may be one strategy to enhance the therapeutic potential of autoimmune and inflammatory diseases such as rheumatoid arthritis and asthma by promoting the induction of immune tolerance [91]. Additionally, only cytokines with known therapeutic effects were examined in this study. It is possible that aligned cell sheets may enhance the secretion of cytokines that are detrimental to therapy. Hence, the secretome requires further comprehensive analysis prior to (pre)clinical applications.

In this study, MSCs from only one donor were used. While secretome profiles are unlikely to change much between donors, the magnitude of the cell patterning/alignment effect on secretome amounts may indeed vary depending on donors, also in harvested sheets. Hence, each donor secretome should be assessed for cell alignment influences in sheets.

MSC sheet detachment performed under ambient temperature reduction yielded detached MSC sheet morphologies shown in Fig. 5C. As reported in many stromal cell sheets, MSC sheet release prompts spontaneous sheet contraction in all cases [82,83,85], but contracted morphological endpoints are distinct: cell-aligned oriented MSC sheets yield detached rectangular shape, whereas non-oriented MSC sheets produce more-circular contracted shapes. Analogous to cell-aligned oriented NHDF sheets (vida supra) aligned and elongated MSCs in oriented sheets contract anisotropically along vectors produced by cytosekeltal alignment prompted by substrate pattern alignment [84], whereas non-oriented MSC sheets do not have a sufficiently strong contractile component alignment and vector, and therefore contract omnidirectionally.

MSC numbers in cultured detached sheets were investigated by dissociation of cell-cell associations by trypsin and subsequent cell counting by trypan blue staining (Fig. 5D). No apparent differences in cell numbers in MSC sheets are observed. Reduced MSC cell numbers are evident in Day 7 sheets than for Day 1 sheets, attributed to difficulties inherent in stable MSC sheet adhesion to patterns in extended culture with cell dissociation and detachment from the sheet and dish surface.

**Fig. 6:**
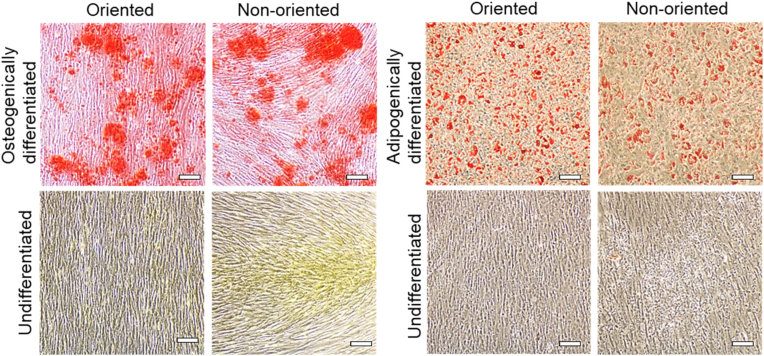
Two-lineage differentiation of oriented MSC sheets. Osteogenic (left 2 columns) and adipogenic (right 2 columns) differentiation was induced for 14 days and confirmed by Alizarin Red S and Oil Red O staining images, respectively. Scale bars: 100 μm.

Multi-potent differentiation ability of MSCs in sheets (i.e., osteogenic and adipogenic differentiation) was conducted by sheet cultures in both osteogenic and adipogenic differentiation media (Fig. 6 and Fig. S2). Cell-aligned oriented and non-oriented MSC sheets were similarly positive for osteogenic Alizarin Red S and Oil Red O staining, indicating that cell alignment in adherent cultured MSC sheets has little observable alterations in their osteogenic and adipogenic differentiation abilities compared to non-aligned MSC sheets.

## 4 Conclusions

Overall, these new findings support the utility of patterned thermos-responsive culture surfaces to fabricate anisotropic, oriented NHDF and MSC sheets. Similarities in cell alignment and sheet behaviors for these two cell types in serum-containing cultures can be attributed to the recognized response of their common stromal lineage and phenotypic tendencies to substrate patterning. Cell-aligned oriented NHDF and MSC sheets both manifest common anisotropic alignment in adherent patterned cultures, contractile sheet behaviors when released from substrates, and higher select cytokine secretions than non-aligned sheets. MSC sheets maintain their recognized differentiation capabilities regardless of adherent cell alignment and overall sheet orientation. Thus, oriented stromal (MSC) sheets fabricated using patterned thermo-responsive cultureware are advantageous to yield tissue surrogates and tissue engineering materials with anisotropic structure and that secrete higher amounts of select cytokines. Such aligned cell sheets with reliable multi-lineage potential could improve MSC cell transplantation therapies or tissue engineering applications currently under intense clinical translational focus.

## CRediT authorship contribution statement

**Kenichi Nagase:** Writing – review & editing, Writing – original draft, Methodology, Investigation, Funding acquisition, Conceptualization. **Hasumi Kuramochi:** Methodology, Investigation. **David W. Grainger:** Writing – review & editing, Supervision. **Hironobu Takahashi:** Writing – review & editing, Methodology, Investigation, Conceptualization.

## Electronic supplementary material

Supplementary data is found in the online version of this manuscript.

## Declaration of competing interest

The authors declare that they have no known competing financial interests or personal relationships that could have appeared to influence the work reported in this paper.

## Data Availability

Data are available from the corresponding author upon reasonable request.
