# Supplementary material for: Functional aligned mesenchymal stem cell sheets fabricated using micropatterned thermo-responsive cell culture surfaces
Source: Mater Today Bio. 2025 Mar 10;32:101657. doi: 10.1016/j.mtbio.2025.101657 (PMC11957804; doi:10.1016/j.mtbio.2025.101657)
Supplement: Multimedia component 1 [file mmc1.docx]

*Supplementary Material*

Functional Aligned Mesenchymal Stem Cell Sheets Fabricated Using Micropatterned Thermo-responsive Cell Culture Surfaces

Kenichi Nagase ^a b *^, Hasumi Kuramochi^b^, David W. Grainger^c,d^ and Hironobu Takahashi^e *^

^a^ Graduate School of Biomedical and Health Sciences, Hiroshima University, 1-2-3 Kasumi, Minami-ku, Hiroshima City, Hiroshima, 734-8553, Japan

^b^ Faculty of Pharmacy, Keio University, 1-5-30 Shibakoen, Minato, Tokyo 105-8512, Japan

^c^ Department of Biomedical Engineering, University of Utah, Salt Lake City, Utah 84112 USA

^d^ Cell Sheet Tissue Engineering Center (CSTEC), Department of Molecular Pharmaceutics, University of Utah, Health Sciences, Salt Lake City, Utah 84112 USA

^e^ Institute of Advanced Biomedical Engineering and Science, Tokyo Women’s Medical University, 8-1 Kawada-cho, Shinjuku, Tokyo, 162-8666, Japan

*Corresponding author (Kenichi Nagase)

E-mail: nagase@hiroshima-u.ac.jp

*Corresponding author (Hironobu Takahashi)

E-mail: takahashi.hironobu@twmu.ac.jp

**S.1 Materials**

Acrylamide (AAm), 0.4w/v% trypan blue solution, 70vol% ethanol, 2-propanol, and 4% paraformaldehyde phosphate buffer solution were obtained from Fujifilm Wako Pure Chemicals (Osaka, Japan). 7,7 dimethyl-2,3-dioxobicyclo[2.2.1] heptane-1-carboxylic acid, a water-soluble photo-initiator, camphor quinone, was obtained from Tokyo Chemical Industry (Tokyo, Japan). Commercial temperature-responsive cell culture dishes (UpCell™) were obtained from CellSeed (Tokyo, Japan). Rhodamine-conjugated bovine fibronectin was obtained from Cytoskeleton (Denver, USA). AlexaFluor488-conjugated bovine serum albumin, Dulbecco's Phosphate-Buffered Saline (DPBS (1×)) and penicillin-streptomycin solution were obtained from Thermo Fisher Scientific (Waltham, USA).

Normal human dermal fibroblasts from neonatal sources, fibroblast basal medium, fibroblast growth medium-2, HEPES buffered saline solution, 0.25 mg/mL trypsin/EDTA, trypsin neutralizing solution, human mesenchymal stem cells from bone marrow, mesenchymal stem cell basal medium, mesenchymal stem cell growth medium supplements and growth factors, human mesenchymal stem cell osteogenic differentiation medium were all obtained from Lonza (Basel, Switzerland).

Mesenchymal stem cell adipogenic differentiation medium was obtained from PromoCell (Heidelberg, Germany). Cellbanker™ cell freezing medium was obtained from Zenogen Pharma (Fukushima, Japan). Trypsin (2.5 g/L) / EDTA (1 mmol/L) was obtained from Nacalai Tesque (Kyoto, Japan). ELISA kits for human VEGF, HGF, TGF-beta 1, and IL-6 basic were obtained from R&D Systems (Minneapolis, USA). Alizarin red S and Oil Red O were obtained from Sigma-Aldrich (St. Louis, USA).

**S2. Cell culture**

**S.2.1 NHDF Culture**

Fibroblast culture medium was prepared by adding 10 mL FBS, 5 mL Penicillin-Streptomycin Solution, 0.5 mL insulin, 0.5 mL GA-1000, 0.5 mL hFGF-B to 500 mL of fibroblast basal medium.

The vial of P1 normal human dermal fibroblasts-neonatal cell suspension was rapidly thawed in a 37°C water bath, and cells were seeded into 10cm cell culture dishes and cultured for 24 h in an incubator (37°C, 5% CO_2_). The acclimation medium was removed, 10 mL of fibroblast medium was added, and cells were placed in an incubator (37°C in 5% CO_2_) for 2 days. The acclimation medium was removed and dishes were washed with 10 mL of HEPES-buffered saline. Then 4 mL of 0.25 mg/mL Trypsin/EDTA was added and the mixture was incubated at 25°C for 3 min. Microscopic observation confirmed that approximately 90% of adherent cells detached; 8 mL of trypsin-neutralizing solution was then added, the cell suspension was collected, and cells were centrifuged (220×g, 5 min) at room temperature. Supernatant was removed and 5 mL of cell freezing medium was added to the cell pellet. Cell suspensions were obtained by pipetting and resuspension. Cell suspensions (10 µL) were stained with 10 µL of trypan blue solution and cell count was calculated using a blood cell counting board. Cell count was diluted with cell freezing medium to 5.0×10^5^ cells/mL, dispensed in 1 mL portions, and stored at -196°C. This cell line was used as the cell bank.

Fibroblast medium (10 mL) was added to 10 cm cell culture dishes and incubated (37°C, 5% CO_2_) for 30 min to equilibrate. Vials of P2 neonatal human dermal fibroblast suspension obtained from the cell bank were rapidly thawed in a 37°C water bath, and the resulting cell suspension was added to a 15 mL tube with 5 mL of equilibration medium and centrifuged (220×g for 5 min) at room temperature. Cells were seeded onto 10cm cell culture dishes at 3.5×10^3^ cells/cm^2^ and incubated (37°C, 5% CO_2_) for 2 days.

**S.2.2 MSC Culture**

To 440 mL mesenchymal stem cell basal medium, 50 mL mesenchymal cell growth supplement, 10 mL L-glutamine, 5 mL penicillin-streptomycin solution, 1.5 GA-1000 were added to the mesenchymal stem cell basal medium.

Mesenchymal stem cell media (10 mL) was added to a 10 cm cell culture dish and incubated (37ºC, 5% CO_2_) for 30 min to equilibrate. Vials of P2 human mesenchymal stem cell suspension were rapidly thawed in a 37ºC water bath, 5 mL of the cell suspension was equilibrated, and cells were added to 15mL tubes with medium, and centrifuged (500×g, 5 min) at room temperature; cells were then seeded into 10cm cell culture dishes at 5.0-6.0×10^3^ cells/cm^2^, and incubated (37ºC, 5 % CO_2_) for 2 days.


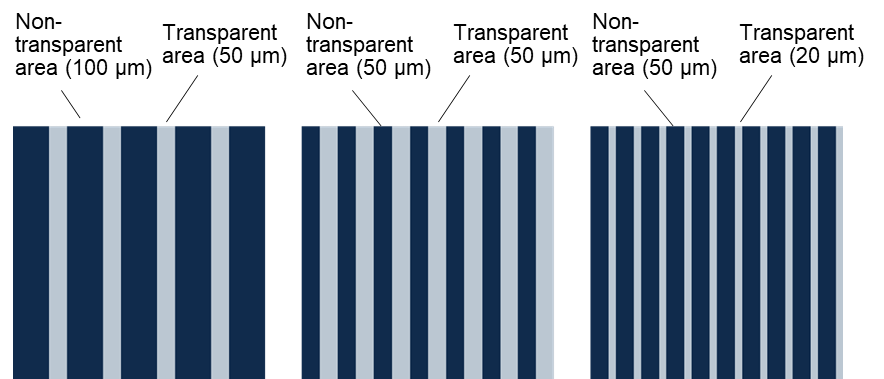


**Figure S1.** Photomask patterns used in this study to fabricate PAAm stripes on PIPPAm commercial thermo-responsive cell culture substrates.


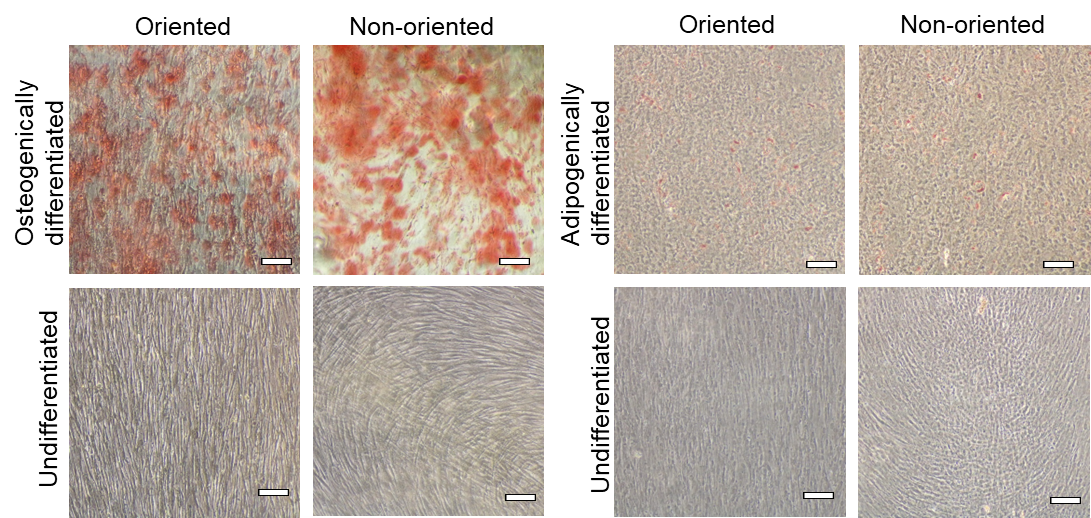


**Figure S2.** Bi-lineage in vitro differentiation capabilities of oriented and non-oriented adherent MSC sheets. Osteogenic (left 2 columns) and adipogenic differentiation (right 2 columns) was performed for 7 days using standard protocols for each differentiation, then stained with Alizarin Red (left images, osteogenic) and Oil Red O (right images, adipogenic), respectively. Scale bars: 100 μm.
